# Exploring the role of shift work in the self-reported health and wellbeing of long-term and assisted-living professional caregivers in Alberta, Canada

**DOI:** 10.1186/s12960-020-00515-6

**Published:** 2020-09-24

**Authors:** Oluwagbohunmi Awosoga, Claudia Steinke, Christina Nord, Jon Doan, Stephanie Varsanyi, Jeff Meadows, Adesola Odole, Sheli Murphy

**Affiliations:** 1 Faculty of Health Sciences (General), University of Lethbridge, 4401 University Drive West Markin Hall M3059, Lethbridge, AB T1K 3 M4 Canada; 2 Faculty of Health Sciences (Nursing), University of Lethbridge, Lethbridge, Alberta Canada; 3 Faculty of Art & Sciences (Department of Psychology), University of Lethbridge, Lethbridge, Alberta Canada; 4 Faculty of Art & Sciences (Kinesiology and Physical Education), University of Lethbridge, Lethbridge, Alberta Canada; 5 Teaching Centre, University of Lethbridge, Lethbridge, Alberta Canada; 6 Department of Physiotherapy, University of Ibadan, Ibadan, Nigeria; 7 Rural Health, Professional Practice, Research and Libraries, Covenant Health, Edmonton, Alberta Canada

**Keywords:** Professional caregivers, Shiftwork, Health status, Wellbeing

## Abstract

**Background:**

Numerous studies have found negative outcomes between shift work and physical, emotional, and mental health. Many professional caregivers are required to work shifts outside of the typical 9 am to 5 pm workday. Here, we explore whether shift work affects the health and wellbeing of long-term care (LTC) and assisted-living (AL) professional caregivers.

**Method:**

The Caring for Professional Caregivers research study was conducted across 39 LTC and AL facilities in Alberta, Canada. Of the 1385 questionnaires distributed, 933 surveys (67.4%) were returned completed. After identifying 49 questions that significantly explained variances in the reported health status of caregivers, we examined whether there was a relationship between these questions and reported health status of caregivers working night shifts.

**Results:**

We found significant differences between responses from those working different shifts across six of seven domains, including physical health, health conditions, mental/emotional health, quality of life, and health behaviors. In particular, we found that night shift caregivers were more likely to report incidents of poor heath (i.e., they lacked energy, had regular presences of neck and back pain, regular or infrequent incidents of fatigue or low energy, had difficulty falling asleep, and that they never do exercise) and less likely to report incidents of good health (i.e., did not expect their health to improve, were not satisfied with their health, do not have high self-esteem/were happy, were unhappy with their physical appearance, and do not get a good night’s sleep), compared to caregivers working other shifts.

**Conclusions:**

Our study shows that professional caregivers working the night shift experience poor health status, providing further evidence that night shift workers’ health is at risk. In particular, caregivers reported negative evaluations of their physical, mental/emotional health, lower ratings of their quality of life, and negative responses to questions concerning whether they engage in healthy behaviors. Our findings can support healthcare stakeholders outline future policies that ensure caregivers are adequately supported so that they provide quality care.

## Background

The demand for professional caregivers in long-term care (LTC) and assisted living (AL) is steadily increasing as the population of seniors requiring care grows [1]. Due to this growing demand, it is important to consider the needs of professional caregivers in order to ensure that they can provide the best care. Furthermore, the needs of professional caregivers can be framed by the physical and mental/emotional demands of their work [2–5]. Using both a quantitative and qualitative approach, the Caring for Paid Professional Caregivers Survey research study explored the health status and quality of life of LTC and AL professional caregivers (hereafter “caregivers”) in the province of Alberta, Canada, as well as factors predicted to influence these health aspect and caregiver dedication to quality care [6]. Additionally, the report provided feasible and relevant strategies that might improve caregivers’ states of health, quality of life, and retention rates in continuing care centers.

The quantitative portion of the survey investigated the physical and mental/emotional health, health conditions, stress, quality of life, and health behaviors (hereafter referred to as “health status”) of LTC/AL caregivers via Likert ratings. Although many caregivers reported that they were healthy during this portion of the survey, their responses to the qualitative portion of the survey revealed another story. In response to open-ended questions and interviews, participants reported sometimes poor health outcomes, such as, “However I believe there are a few main factors that are causing [health] issue[s]. One factor being the heavy workloads, our job as care givers is physically and mentally exhausting and very hard on the body.”; “I work with a chronic injury that was a direct result of an encounter with a resident at my place of work… The pain limits me”; and “Working in L.T.C. is very hard on the body, walking on cement, lifting, twisting, turns. Level of care is very heavy so we need more staffing so we don't have to work so fast to get things done on time.”

As we continued our qualitative analysis, we learned that participants sometimes attributed their poor health outcomes to the stress of shift work. Caregiver responses included calls for employers “…to allow staff to have consistent shifts instead of mixed shifts of days and evenings, and days and nights which is making staff have difficulty sleeping which lead to other medical issues such as migraines [sic] and other illness, stemming from a lack of sleep;” as well as for employers to “…[allow] staff to work the shifts that fit into their life whether it be straight days or evenings or nights with only required as per contract days.”

Shift work, or work outside of the typical 9 am to 5 pm workday, is known to have physical, emotional, and mental effects [7, 8]. In healthcare industries, shift work usually encompasses either a three-shift system, with day, evening, and night shifts each lasting 8 h, or a rotating shift system [9]. Shift work requires workers to function when it is dark and sleep during the day, in direct opposition to the typical human circadian cycle [7]. This results in disturbed circadian rhythms, characterized by disturbed sleeping patterns along with less sleep overall compared to non-shift workers [10, 11]. These disturbed sleeping patterns are in turn associated with various forms of neurocognitive decline, such as lapses of attention, increased time it takes to react, and increased errors, which can be apparent in one’s work (e.g., work errors and injuries) [11].

As of 2016, shift workers comprised 15–25% of global workforce [12]. Shift work is increasingly recognized as an important area for research [13]. Sleep disruption in healthcare workers is a worsening problem, with workers who report 6 or less hours of sleep per night increasing from 28% between 1985 and 1990 to 32% between 2004 and 2007 [14]. Problems from shift work are especially prevalent in healthcare work due to its demanding schedules that require caregivers to sleep at times that are out of phase with typical circadian rhythms [14]. Such sleep disruption can result in errors in the delivery of patient care [14]. Additionally, the requirements of shift work and the problems associated therein remain a major reason nurses leave the nursing profession [14].

At the extreme end, individuals working shift schedules are susceptible to shift work disorder (SWD), prevalent in 2–5% of the US working populations [15]. Currently, there are two important risk factors for SWD that are also highly correlated with the professional caregiving population. The first is that caregivers are overwhelmingly female, and females appear to be particularly susceptible to SWD and suffer more injuries associated with shift work [16, 17]. The second is that the caregiving population is also aging, with 50% of Canadian professional caregivers aged between 45 and 65, and older age correlates with increased risk of impaired sleep quality and a greater risk of excessive sleepiness in shift workers [13, 18].

Thus, the qualitative responses to our survey, as well as the established impact of shift work on physical and mental/emotional health, inspired us to investigate whether or not there was a relationship between shift work and health status in professional caregivers. Using responses to the quantitative portion of the Caring for Paid Professional Caregivers research study [6], we explored the possibility that LTC/AL caregivers working the night shift report experiencing poorer physical health, health conditions, mental/emotional health, quality of life, and health behaviors compared to caregivers on other shifts. Understanding whether or not there is a relationship between shift work and health in the LTC/AL population is important for caregivers, their employers, and policy makers so that the adverse consequences associated with shift work can be not only addressed, but also prevented.

## Methods

### Caring for Paid Professional Caregivers research study

The Caring for Paid Professional Caregivers research study [6] was conducted across 39 LTC and AL facilities across Alberta. Using both a quantitative and qualitative approach, we designed this survey to explore factors that influence caregivers’ health status, quality of life, and dedication to quality care in continuing care settings. Thus, data collected from this survey instrument included responses to questions that probed the physical health, health conditions, mental/emotional health, stress, quality of life, health behavior, turnover and absenteeism, and demographic information of caregivers, alongside two qualitative questions exploring how caregivers thought their employers could support and improve caregiver health statuses. There was also a follow-up interview with seven participants to further explore these questions.

To conduct the survey, the principal investigator (PI) compiled a list of continuing care centers across Alberta identified as targets for study. The PI then contacted the Alberta Health Services Research Committee for approval of this list and to contact site administrators. After centers agreed to participate, questionnaires were given to managers at each center in sealed envelopes and included return envelopes so that respondents could easily conceal their responses. Managers placed the surveys into all staff mailboxes along with a $5 gift card as honorarium. A secured drop-box was placed into staff break rooms for caregivers to deposit completed surveys. For the follow-up interview portion of the study, email communication was substituted for the originally proposed face-to-face design interview due to time constraints and because participation interest was lower than initially indicated.

In all, 1385 questionnaires were sent to LTC and AL facilities, and 933 surveys (67.4%) were returned with completed responses. For these analyses, we compared the health status of caregivers who worked the night shift to those who worked either the day shift, evening shift, or some combination of day, evening, and night shifts, which we refer to as a “rotating shift.” Additionally, while these analyses were inspired by the qualitative portion of our survey, we used only responses to the quantitative portion in our analyses here.

### Survey question selection

For the purposes of this study, we selected the 49 questions (of 98 total questions) from the quantitative portion of the Caring for Paid Professional Caregivers research study [6] that were found to significantly explain variances in the reported health status (i.e., physical health, health conditions, mental/emotional health, stress, quality of life, and health behavior) of long-term care and assisted living caregivers (see Supplementary Table S1) [6]. We then used these 49 questions to investigate whether there was a relationship between shift work and the health status of professional caregivers.

### Statistical analysis

#### Dataset adjustment

In our analyses, our independent variable was shift worked (either day shift, evening shift, night shift, or a rotating shift). In order to increase the probability that the assumptions of statistical tests would be met, we first adjusted the survey dataset by combining similar response categories for a few of the questions. All response options that were adjusted are indicated by a “/” separating the combined response options in Table 2. In particular, for questions regarding physical health, we combined the response options of “strongly agree” and “agree”, as well as “strongly disagree” and “disagree”. For questions concerning heath conditions, we combined the response options of “regular/often” and “persistent” (note: “regular/often was an original response option), as well as “sometimes” and “infrequently.” For questions exploring mental/emotional health questions, we combined the response options of “strongly agree” and “agree,” as well as “strongly disagree” and “disagree.” For questions concerning stress, we combined the responses of “high” and “very high,” as well as “low” and “medium.” Responses to questions regarding quality of life were not combined. Finally, for questions exploring health behavior, we combined the response options of “regular/often” and “persistent” (note: “regular/often was an original response option), as well as “sometimes” and “infrequently.” For the sake of brevity, we refer to the less-extreme response in these combined responses when discussing the results, discussions, and conclusions. For example, responses of “regular/often/persistent” are referred to as responses of “regular.”

#### Chi-square test for independence

To examine the relationship between reports of health status and the shifts caregivers worked, we performed a chi-square test of independence for each question found to significantly explain variances in caregiver reports of health status, reported in Supplementary Table S1 using Statistical Package for Social Sciences software version 25 [19]. However, 13 of the 49 questions did not meet the assumptions of the test (expected counts were less than 5). Of the 36 questions remaining, 14 were found to have significant differences between groups concerning shifts worked and reports of health status (and 22 found to have no significant differences between groups; Table 2).

#### *Z*-tests

Finally, we performed *z*-tests using Statistical Package for Social Sciences (SPSS) [19] on the 14 questions found to have significant differences between reports of health status and shifts worked (Table 2). We used Bonferroni correction to determine the level of significance because we made pairwise comparisons.

## Results

Of these 14 questions, 12 revealed significant differences between the health status of those working the night shift compared to those working other shifts (Table 2).

### Participants

Participant demographics are presented in Table 1. Overall, our sample reflects the Albertan caregiving workforce at large in regard to gender (overwhelmingly female [17]), as well as in age distribution (see below) and nature of appointment [20, 21].

**Table 1:** Demographics of caregiver participants

Demographic	***N***	Percentage
**Gender**		
Female	833	90.7
Male	83	9.0
Non-binary	1	0.1
Unsure	1	0.1
**Age**		
18–25	74	8.1
26–34	168	18.3
35–44	228	24.9
45–54	224	24.4
55+	223	24.3
**First language**		
English	644	72.5
Not English	244	27.5
**Shift worked**		
Day	293	32.0
Evening	49	5.3
Night	29	3.2
Unsure	2	0.2
Rotating	544	59.3
**Hours worked per week**		
< 20 h	62	6.8
20–40 h	615	67.3
41–60 h	205	22.4
> 60 h	32	3.5
**Nature of appointment**		
Full-time	328	35.4
Part-time	494	53.3
Casual	101	10.9
Unsure	4	0.4
**Position**		
Registered nurse (RN)	121	13.2
License practical nurse (LPN)	114	12.4
Health care aids (HCA)	534	58.1
Other staff^a^	150	16.3
**Experience**		
0–2 years	129	14.1
2–5 years	201	22.0
5–10 years	218	23.9
10–20 years	218	23.9
20+ years	148	16.2
**Education**		
Less than a high school diploma	27	3.0
High school diploma	161	17.6
College diploma^b^	421	46.1
Bachelor/graduate degree	252	27.6
Others	52	5.7
**Geographic zone**		
North	50	5.4
Edmonton	172	18.4
Central	207	22.2
Calgary	122	13.1
South	382	40.9

^a^e.g., occupational therapists, social workers, physiotherapists, dietitians

^b^Two-year associate degrees or 4-year applied degree

In our sample, most participants reported working rotating shifts (59.3%). Day shift workers made up 32.0% of all participants, with evening shift workers making up 5.3%, and night shift workers comprising of 3.2%.

Most participants were 35 years or older (73.6%), with a significant portion above 45 years of age (48.7%). Most participants were female (90.7%). A majority of participants reported that English was their first language (72.5%), contrasting with prior work indicating that only 48.8% of Albertan caregivers speak English as a first language [20].

A majority of participants reported working 20–40 h per week (67.3%), and 22.4% reported working 41–60 h. A small percentage reported working less than 20 h a week (6.8%), and only 3.5% reported working more than 60 h a week. Furthermore, most participants reported working on a part-time basis (53.3%), with the next largest percentage comprising full-time employees (35.4%). Casual employees made up 10.9% of participants.

The majority of participants were health care aids (HCA, 58.1%), followed by other staff (e.g., occupational therapists, social workers, physiotherapists, dietitians; 16.3%), registered nurses (RN, 13.2%), and licensed practical nurses (LPN, 12.4%). Experience in LTC/AL was spread widely across the response options ranging from less than a year to more than 20 years. Those working in LTC/AL for 0–2 years comprised 14.1% of the sample, and those working in LTC/AL for 2–5 years comprised 22.0%. The percentage of participants working for 5–10 years in LTC/AL equaled those working for 10–20 years (23.9%). In addition, those working 20 years or more in LTC/AL comprised 16.2% of the sample.

Only 3.0% of participants reported having less than a high school diploma. High school diploma recipients made up 17.6% of participants. The majority of participants reported having a college diploma (i.e., 2-year associate degrees or 4-year applied degree; 46.1%), and a significant portion reported attaining a bachelor or graduate degree (27.6%).

Finally, our sample was comprised of caregivers who worked across the Alberta province; 39 LTC and AL facilities participated in this study across five geographic regions of Alberta. The majority of participants (40.9%) came from the south, and 22.2% were located in the center of the province. The two largest cities, Edmonton and Calgary, made up 18.4% and 13.1% of participants, respectively. Finally, 5.4% of the participants came from the north of the province. For these analyses, we combined the participants from these areas to create one dataset.

### Physical health

In terms of physical health, we found significant differences between the responses of those working different shifts using the chi-square analysis (3 of 7 questions; an additional question was not significant pairwise due to the Bonferroni adjustment; see Table 2). *Z*-tests revealed that caregivers working the night shift were more likely to report that they do not have a lot of energy, were less likely to agree that they expect their health to improve, and were less likely to agree that they are satisfied with their health compared to those working day, evening, or rotating shifts (and responded to this question with significantly more neutrality than those working day or rotating shifts; Table 2).

**Table 2:** Results of pairwise comparisons between groups’ post hoc analyses for questions found to have a significant difference between groups in the chi-square analysis

Category	Question	***Pearson X***^**2**^ (6)	Cramer’s V (Approx. Sig)	Response options	***Comparisons of column proportions (z-test)***			
					Day Shift	Evening shift	Night shift	Rotating shift
Physical health	I feel I have a lot of energy (*n* = 912)	29.098	.179 (.000)	Strongly disagree/disagree	34 (11.6%)	7 (14.3%)	*13 (44.8%)* Day**** Evening* Rotating****	68 (12.5%)
				Neutral	80 (27.4%)	8 (16.3%)	5 (17.2%)	154 (28.4%)
				Strongly agree/agree	178 (61.0%)	*34 (69.4%)* Day*	11 (38.0%)	320 (61.1%)
Physical health	I expect my health to get better (*n* = 896)	15.052	.092 (.020)	Strongly disagree/disagree	33 (11.5%)	3 (6.3%)	6 (22.2%)	59 (11.0%)
				Neutral	87 (30.3%)	11 (22.9%)	14 (51.9%)	163 (30.5%)
				Strongly agree/agree	*167 (58.2%)* Night**	*34 (70.8%)* Night***	7 (25.9%)	*312 (58.4%)* Night**
Physical health	Overall, I am satisfied with my health (*n* = 909)	15.430	.092 (.017)	Strongly disagree/disagree	30 (10.3%)	3 (6.1%)	5 (17.2%)	69 (12.8%)
				Neutral	64 (21.9%)	10 (20.4%)	*13 (44.8%)* Day* Rotating**	106 (19.7%)
				Strongly agree/agree	*198 (67.8%)* Night**	*36 (73.5%)* Night*	11 (37.9%)	*364 (67.5%)* Night**
Health conditions	Presence of neck /back ache (*n* = 916)	22.714	.111 (.001)	Regular/often/persistent	79 (27.0%)	15 (30.6%)	*16 (55.2%)* Day**	*196 (36.0%)* Day*
				Sometimes/infrequently	*194 (66.2%)* Night*	25 (51.0%)	12 (41.4%)	311 (57.1%)
				Never	20 (6.8%)	*9 (18.4%)* Day* Rotating*	1 (34.4%)	38 (7.0%)
Health conditions	Incidence of fatigue or low energy (*n* = 909)	12.713	.084 (.048)	Regular/often/persistent	63 (21.6%)	12 (24.5%)	*14 (48.3%)* Day** Rotating*	123 (22.8%)
				Sometimes/infrequently	*204 (70.1%)* Night**	35 (71.4%)	12 (41.4%)	*371 (68.7%)* Night*
				Never	24 (8.2%)	2 (4.1%)	3 (10.3%)	46 (8.5%)
Mental/emotional heath	I have high self-esteem/feel happy with myself, I am a happy person (*n* = 917)	14.238	.088 (.027)	Strongly disagree/disagree	15 (5.1%)	2 (4.1%)	5 (17.2%)	33 (6.0%)
				Neutral	54 (18.4%)	6 (12.2%)	9 (31.0%)	120 (22.0%)
				Strongly agree/agree	*224 (76.5%)* Night*	*41 (83.7%)* Night*	15 (51.7%)	393 (72.0%)
Mental/emotional heath	I have a good level of motivation (*n* = 915)	16.637	.095 (.011)	Strongly disagree/disagree	8 (2.7%)	0 (0.0%)	3 (10.3%)	25 (5.0%)
				Neutral	48 (16.4%)	8 (16.3%)	*11 (38.0%)* Day* Rotating*	98 (18.0%)
				Strongly agree/agree	*237 (80.9%)* Night**	*41 (83.7%)* Night*	15 (51.7%)	*421 (77.4%)* Night**
Mental/emotional heath	I have difficulty falling or staying asleep (*n* = 912)	14.622	090 (.023)	Strongly disagree/disagree	*147 (50.1%)* Night*	*28 (58.3%)* Night**	6 (21.1%)	*253 (46.5%)* Night*
				Neutral	45 (15.5%)	9 (18.8)	10 (34.5%)	98 (18.0%)
				Strongly agree/agree	99 (30.4%)	11 (22.9%)	13 (44.8%)	193 (35.5%)
Quality of life	Your physical appearance (*n* = 916)	23.572	.093 (.023)	Terrible	3 (1.0%)	1 (2.1%)	*3 (10.3%)* Day**	14 (2.6%)
				Unhappy	28 (9.6%)	1 (2.1%)	3 (10.3%)	40 (7.3%)
				Mixed	83 (28.3%)	9 (18.8%)	9 (31.0%)	134 (24.5%)
				Satisfied	109 (37.2%)	18 (37.5%)	11 (38.0%)	221 (40.5%)
				Happy	70 (23.9%)	*19 (39.6%)* Night*	3 (10.3%)	137 (25.1%)
Heath behavior	I do exercise three times a week for at least 20 minutes each time (*n* = 917)	14.221	.088 (.027)	Never	23 (7.8%)	5 (10.2%)	*8 (27.6%)* Day** Rotating*	58 (10.6%)
				Sometimes/infrequently	154 (52.6%)	22 (44.9%)	13 (44.8%)	298 (54.6%)
				Regular/often/persistent	116 (39.6%)	22 (44.9%)	8 (27.6%)	190 (34.8%)
Heath behavior	I get a good sleep at night (7-8 hours of sleep) (*n* = 912)	31.503	.131 (.000)	Never	25 (8.6%)	8 (16.3%)	6 (20.7%)	60 (11.0%)
				Sometimes/infrequently	134 (46.2%)	14 (28.6%)	*20 (69.0%)* Evening**	*301 (55.3%)* Evening**
				Regular/often/persistent	*131 (45.2%)* Night** Rotating**	*27 (55.1%)* Night*** Rotating*	3 (10.3%)	183 (33.6%)
Heath behavior	I visit my doctor for routine checkup (*n* = 914)	13.116	.085 (.041)	Never	18 (61.6%)	3 (6.1%)	2 (6.9%)	36 (66.2%)
				Sometimes/infrequently	95 (32.5%)	20 (40.8%)	11 (37.9%)	*244 (44.9%)* Day**
				Regular/often/persistent	*179 (61.3%)* Rotating**	26 (53.1%)	16 (55.2%)s	264 (48.5%)

Note: Results are based on two-sided *z*-tests. Cells in italics indicate significant differences between groups. For each significant pair, the group with the smaller proportion is indicted in the column of the group with the larger column proportion. For example, in the first row, caregivers’ working day, evening, or day/evening/night shifts answered “Strongly disagree/disagree” to the question “I feel that I have a lot of energy” significantly fewer times than those working the night shift. Shift types and responses to questions were categorized by how caregivers responded these specific questions in the survey. Significance level: *.05., **.01, ***.001, ****.00001

### Health conditions

The chi-square analysis revealed significant differences between shifts worked and questions concerning health conditions (2 of 9 questions; an additional question was not significant pairwise due to the Bonferroni adjustment; see Table 2). The *z-*tests revealed that compared to caregivers working the day shift, night shift caregivers were more likely to report regular presence of neck and back aches (and less likely to never experience such pain compared to those working evening shifts), and more likely to report regular or infrequent incidents of fatigue or low energy compared to those working day and rotating shifts.

### Mental/emotional health

In terms of the mental/emotional health of caregivers, the chi-square analysis revealed significant differences between shifts worked (3/12 questions, Table 2). Using a *z*-test, we found that caregivers working the night shift were less likely to report that they have high self-esteem/are happy compared to those working day or evening shifts, good levels of motivation compared to those working all other shifts, and less likely to disagree that they have difficulty falling asleep compared to those working all other shifts.

### Stress

Of the five questions that probed caregiver stress levels, the chi-square analysis found no significant differences between those working different shifts (Supplementary Table 1).

### Quality of life

For responses to questions exploring caregivers’ ratings of their quality of life, the chi-square analysis found a significant difference between shifts worked (1 of 8 questions; Table 2). In particular, the *z*-tests revealed that caregivers working the night shift were less likely to report that they were happy with their physical appearance compared to those working the evening shift (and more likely to report that they feel terrible with their physical appearance compared to those working the day shift, Table 2).

### Health behaviors

In terms of health behaviors, the chi-square analysis revealed significant differences between shifts worked (3 of 8 questions, Table 2). The *z*-tests revealed that caregivers working the night shift were more likely to report that they never do exercise compared to those working the day or rotating shift, and were less likely to report that they get a good sleep at night compared to those working the day or evening shift. We found no significant differences between those working the night shift and those working other shifts in their likelihood to visit their doctor for checkups (Table 2).

## Discussion

In our study, we found that caregivers working the night shift report poorer health statuses compared to those working other shifts. Many of these poor outcomes are either directly or indirectly related to the requirements of their work, including physical and emotional demands and a lack of adequate rest periods. Similar studies have found that health care workers are particularly vulnerable to fatigue and sleep-related disorders and that fatigue can have an important mediating effect between quality of life and mental health [13, 22]. Given these findings, it is important that caregivers are adequately supported by their employers to ensure that they can provide the best care, and preventing SWD and the deleterious effects of shift work is a shared responsibly between caregivers and employers. For example, employers determine whether there is adequate time for caregivers to sleep between shifts, and it is up to caregivers to be rested before coming to work [23]. Employers and managers of LTC/AL facilities should be aware and appreciative of the demands of shift work, its associated long work hours, and its costs [14]. There is evidence that employees experience more positive outcomes when they are allowed input, choice, and flexibility in their work schedules [15, 24–26]. While collective bargaining agreements offer a potentially productive area in which professional caregivers could negotiate stipulations regarding shift work, examples of such are few beyond establishing shift-related wage differentials [27]. Additionally, professional caregivers often lack access to unionization [28, 29].

Fortunately, there are many strategies to mitigate the costs of shift work, as well as to prevent SWD. Zhang et al. [30] found that for each unit increase in a number of beneficial work environment features, the prevalence of short sleep durations decreased by 7%, and the prevalence of poor sleep quality decreased by 17%. Such features included the presence of a work environment that had low physical demands and high physical safety, as well as low incidents of violence at work, low psychological demands of caregivers, high decision latitude of caregivers, high social support of caregivers, and low work-family conflict in caregivers [30]. These findings are particularly relevant to our findings that professional caregivers working the night shift experience greater physical demands, and higher incidences of poor sleep quality, compared to caregivers working other shifts.

Professional caregiving is often very physically demanding, which can result in injury [2, 3]. Such demands can increase the possibility of accidents [14]. We found that 55.2% of caregivers working night shifts experience regular presence of neck and back aches, and this was significantly more than the 27.0% of day shift workers who had similar complaints. In this regard, Caruso [14] suggests that management provide an anonymous, no-blame reporting system of workplace accidents in order to determine if fatigue has contributed. This is especially important considering the clear safety concerns with working the night shift that stem from fatigue, as well as often having less supervision and support compared to non-daytime shifts. We also found that professional caregivers working the night shift are experiencing fatigue, low energy, have difficultly falling asleep, and do not get a good night’s sleep. Using the Survey of Labour and Income Dynamics, Wong et al. [16] found that while the rate of overall injuries among Canadian workers decreased from 1996 to 2006, this was not the case for night shift workers. Women in particular were found to have a 14.4% risk of injury compared to 8.2% for men. This reveals a particularly salient risk to the women-dominated workforce of LTC/AL.

The Caring for Paid Professional Caregivers research study also includes strategies that can also help to mitigate the cost of shift work [6]. These strategies include improving work schedules, allowing employees to take frequent breaks, promoting positive relationships both between caregivers themselves and between caregivers and their supervisors, and creating policies that support shift workers [6, 14]. The Canadian Centre for Occupational Health and Safety recommends that organizations implement a “shift schedule design” which suggests specific rotation period lengths, directing rotating shifts forward from day to afternoon to night, having at least 24 h of rest between sets of night shifts, and allowing alternative forms of work schedule organization, such as extending shifts to 10 or 12 h to reduce the number of consecutive night shifts (but caution that extended shifts do increase physical and mental load) [31]. Additionally, it is recommended that shift workers be informed of their shifts ahead of time so that they can plan activities with families and friends, thereby allowing them an opportunity to offset the social disadvantages of night shift work [31]. The Canadian Centre for Occupational Health and Safety also recommends actions specific to organizations design, including ensuring that there is adequate lighting and ventilation on all shifts, providing rest facilities whenever possible, providing healthy cafeteria services in order to encourage a balanced diet, and educating employees about the health and safety effects of shift work [31].

## Limitations

Our study is limited by the fact that participants’ recruitment was focused only on LTC/AL facilities owned by Alberta Health Services and Covenant Health located in Alberta, Canada, and was only provided in English. Additionally, few private (for-profit/not-for-profit) LTC/AL providers and agencies were also included in this study. Our study also is also limited by that fact that very few participants (3.2%) worked the night shift only and that the initially proposed face-to-face follow-up interviews were discontinued and replaced with email communication due to time constraints, and because participation interest was lower than initially indicated. Finally, our cross-sectional design makes it difficult to completely evaluate the causal relationship between shifts worked and causal variables.

## Conclusions

We found evidence that caregivers working the night shift report lower health statuses compared to caregivers working other shifts in LTC/AL facilities across Alberta. When we explored questions that predicted the health of caregivers, we found some evidence that those working the night shift report more negative evaluations of their physical and mental/emotional health, lower ratings of their quality of life, and negative responses to questions concerning healthy behaviors. In particular, of the 12 questions in which we found differences between reports of the health status of caregivers and shift schedules, 11 revealed response indicated poor health status of caregivers working the night shift compared to those working other shifts. Thus, despite an overall finding that LTC and AL caregivers across Alberta reported having positive health statuses [6], those working the night shift reported poorer heath statuses than those working other shifts. This finding presents a potential risk to the health and wellbeing of LTC and AL caregivers working night shifts. Therefore, the potential impacts of shift work should be considered in any future research investigating the health of workers in shift work-dependent fields.

Currently, there are no occupational exposure limits for night shift work in Canada, but there are various organizations committed to protecting caregivers working night shift, including the Canadian Centre for Occupational Health and Safety, The Workers Health and Safety Centre, and The Institute for Work and Health. Taking the risks of shift work seriously in LTC/AL both prevents SWD and other adverse outcomes associated with shift work, including burnout. Shift work is an occupational risk for burnout syndrome (BOS) in health care workers [32]. Importantly, BOS develops gradually. Such gradual development has the potential to afford early intervention and prevention of full BOS. Therefore, organizations that take both preventative measures to prevent the adverse outcomes of shift work and that address the problems associated with shift work have the potential to not only decrease the development of SWD and burnout among their employees, but also ensure that caregivers can provide the best care to their clients.

## Supplementary information

**Additional file 1: Table S1.** Questions from the Caring for Paid Professional Caregivers Survey [6] found to significantly explain variances in the reported health status of long-term care and assisted living caregivers.

## Data Availability

The datasets used and/or analyzed during the current study are available from the corresponding author (Dr. Oluwagbohunmi Awosoga) on reasonable request.

## Abbreviations

LTC: Long-term care
AL: Assisted living
SWD: Shift work disorder
PI: Principle investigator
HCA: Health care aide
RN: Registered nurse
LPN: Licensed practical nurse
BOS: Burnout syndrome
