# Supplementary material for: Exploring the role of shift work in the self-reported health and wellbeing of long-term and assisted-living professional caregivers in Alberta, Canada
Source: Hum Resour Health. 2020 Sep 24;18:70. doi: 10.1186/s12960-020-00515-6 (PMC7517821; doi:10.1186/s12960-020-00515-6)
Supplement: Supplementary file 1 — Additional file 1: Table S1. Questions from the Caring for Paid Professional Caregivers Survey [6] found to significantly explain variances in the reported health status of long-term care and assisted living caregivers. [file 12960_2020_515_MOESM1_ESM.docx]

| **Category** | **Question (n)** | ***Pearson X*^2^ (6)** | **# cells with expected count <5 (%)** | **Asymptotic Significance (2-sided)** | **Cramer’s V (Approx. Sig)** |
| --- | --- | --- | --- | --- | --- |
| **Physical Health** | In comparison with people of your age, how would you rate your own state of health? (n = 856) | 6.886 | 3 (25.0%) | 0.331 | .063 (.331) |
|  | *I feel I have a lot of energy (n = 912)* | *29.098* | *1 (8.3%)* | *0.000* | *.179 (.000)* |
|  | I am free of chronic disease (n = 909) | 6.394 | 3 (25.0%) | 0.381 | .059 (.381) |
|  | *I expect my health to get better (n = 896)* | *15.052* | *1 (8.3%)* | *0.020* | *.092 (.020)* |
|  | I am healthy as anybody I know * (n = 914) | 10.195 | 1 (8.3%) | 0.117 | .075 (.117) |
|  | I seem to get sick a little easier than other people (n=902) | 13.028 | 2 (16.7%) | 0.043 | .085 (.043) |
|  | *Overall, I am satisfied with my health (n = 909)* | *15.430* | 1 (8.3%) | *0.017* | *.092 (.017)* |
| **Health Conditions** | *Presence of neck /back ache (n = 916)* | *22.714* | *2 (16.7%)* | *0.001* | *.111 (.001)* |
|  | Presence of sore arms/legs * (n = 916) | 14.643 | 1 (8.3%) | 0.023 | .089 (.023) |
|  | Feeling of tension, stiffness, or lack of flexibility in your spine (n = 912) | 1.793 | 0 (0.0%) | 0.938 | .031 (.938) |
|  | *Incidence of fatigue or low energy (n = 909)* | *12.713* | *2 (16.7%)* | *0.048* | *.084 (.048)* |
|  | Incidence of colds/flu or cough (n = 914) | 4.632 | 4 (33.3%) | 0.592 | .050 (.592) |
|  | Incidence of headaches (n = 906) | 7.551 | 1 (8.3%) | 0.273 | .065 (.273) |
|  | Incidence of breathless with slight exertion (n = 908) | 4.900 | 2 (16.7%) | 0.557 | .052 (.557) |
|  | Presence of Hypertension (n = 912) | 3.854 | 2 (16.7%) | 0.696 | .046 (.696) |
|  | Presence of Diabetes (n = 912) | 7.201 | 4 (33.3%) | 0.303 | .063 (.303) |
| **Mental and Emotional Health** | *I have high self-esteem/feel happy with myself, I am a happy person (n = 917)* | *14.238* | *2 (16.7%)* | *0.027* | *.088 (.027)* |
|  | I am comfortable with ‘negative’ emotions (sadness, guilt, anger, envy) (n = 907) | 4.917 | 0 (0.0%) | 0.554 | .052 (.554) |
|  | I feel excited to be alive when I wake up in the morning (n = 915) | 12.255 | 2 (16.7%) | 0.057 | .082 (.057) |
|  | *I have a good level of motivation (n = 915)* | *16.637* | *2 (16.7%)* | *0.011* | *.095 (.011)* |
|  | I feel compassion for my work (n = 911) | 12.163 | 4 (33.3%) | 0.058 | .082 (.058) |
|  | I feel a sense of belonging with where I am (n = 907) | 7.616 | 3 (25.0%) | 0.268 | .065 (.268) |
|  | I am able to handle fear and anxiety (n = 912) | 12.326 | 2 (16.7%) | 0.055 | .082 (.055) |
|  | I feel I am calm and filled with inner peace, tranquility, and peace of mind (n = 914) | 7.157 | 1 (8.3%) | 0.307 | .063 (.307) |
|  | I have negative or critical feelings about myself (n = 913) | 9.702 | 0 (0.0%) | 0.138 | .073 (.138) |
|  | *I have difficulty falling or staying asleep (n = 912)* | *14.622* | *0 (0.0%)* | *0.023* | *090 (.023)* |
|  | I experience recurring thoughts or dreams (n = 903) | 7.667 | 2 (16.7%) | 0.264 | .065 (.264) |
|  | I am emotionally healthy (n = 915) | 7.326 | 1 (8.3%) | 0.292 | .063 (.292) |
| **Stress** | Family (n = 912) | 4.482 | 2 (16.7%) | 0.612 | .050 (.612) |
|  | Work (n = 910) | 3.512 | 2 (16.7%) | 0.742 | .044 (.742) |
|  | Finance (n = 909) | 10.791 | 1 (8.3%) | 0.095 | .077 (.095) |
|  | General Status (n = 908) | 4.262 | 2 (16.7%) | 0.641 | .048 (.641) |
|  | Emotional Status (n = 914) | 7.623 | 2 (16.7%) | 0.267 | .065 (.267) |
| **Quality of Life** | Your personal life (n = 914) | 8.941 | 6 (30.0%) | 0.708 | .057 (.708) |
|  | Your significant other /spouse /partner (n = 866) | 6.568 | 6 (30.0%) | 0.885 | .050 (.885) |
|  | Your financial needs (n = 916) | 13.094 | 3 (15%) | 0.362 | .069 (.362) |
|  | Your co-workers (n = 914) | 15.010 | 5 (25.0%) | 0.241 | .074 (.241) |
|  | Your handling of problems in your life (n = 907) | 20.661 | 6 (30.0%) | 0.056 | .087 (.056) |
|  | What you are actually accomplishing (n = 913) | 9.439 | 6 (30.0%) | 0.665 | .059 (.665) |
|  | *Your physical appearance (n = 916)* | *23.572* | *4 (20%)* | *0.023* | *.093 (.023)* |
|  | Your health (n = 914) | 17.128 | 6 (30.0%) | 0.145 | .079 (.145) |
| **Health Behaviour** | I always eat a diet high in fiber (n = 914) | 7.880 | 2 (16.7%) | 0.247 | .066 (.247) |
|  | I try to avoid eating high fat foods (n = 916) | 5.498 | 2 (16.7%) | 0.482 | .055 (.482) |
|  | *I do exercise three times a week for at least 20 minutes each time (n = 917)* | *14.221* | *1 (8.3%)* | *0.027* | *.088 (.027)* |
|  | I drink adequate amounts of fluids (1/2 ounce per pound body weight) (n = 914) | 11.245 | 3 (25.0%) | 0.081 | .078 (.081) |
|  | I follow the Canadian food guide (7-10 servings of vegetables and fruit, 2-3servings of meat, and 2 servings of milk) (n = 915) | 5.548 | 2 (16.7%) | 0.476 | .055 (.476) |
|  | I am at the ideal body weight for someone my height (n = 909) | 7.057 | 1 (8.3%) | 0.316 | .062 (.316) |
|  | *I get a good sleep at night (7-8 hours of sleep) (n = 912)* | *31.503* | *1 (8.3%)* | *0.000* | *.131 (.000)* |
|  | *I visit my doctor for routine checkup (n = 914)* | *13.116* | *2 (16.7%)* | *0.041* | *.085 (.041)* |

*Note: Rows in italics indicate questions found to have differences between groups in the chi-square analyses.* Indicates comparisons that were not significant pairwise because of the Bonferroni adjustment.*
